# Identifying performance indicators to measure overall performance of telephone triage – a scoping review

**DOI:** 10.1080/02813432.2023.2283188

**Published:** 2024-02-07

**Authors:** Hanna Vainio, Leena Soininen, Maaret Castrén, Paulus Torkki

**Affiliations:** a Department of Emergency Medicine and Services, Helsinki University and Helsinki University Hospital, Helsinki, Finland; b DigiFinland Ltd., University of Helsinki, Helsinki, Finland; c Emergency Medicine, Department of Emergency Medicine and Services, Helsinki University and Helsinki University Hospital, Helsinki, Finland; d Department of Public Health, University of Helsinki, Helsinki, Finland

**Keywords:** Emergency medical services, primary healthcare, organizational performance, performance measurement, telephone triage

## Abstract

**Objective:**

This article aims to summarize performance indicators used in telephone triage services research, and make recommendations for the selection of valid indicators to measure the performance of telephone triage. We describe what kind of frameworks, performance indicators, or variables have been used for evaluating telephone triage performance by systematically mapping the telephone triage performance measurement. The objective was to find measures for each Triple Aim dimension.

**Design:**

A scoping review method was used following Joanna Briggs Institute guidelines. Using this method, we defined indicators to measure the performance of telephone triage. We used the Triple Aim framework to identify indicators to measure the overall performance of telephone triage. The Triple Aim framework consists of improving the patient experience of care, improving the health of populations, and reducing cost per capita.

**Setting:**

The scoping review was performed using CINAHL, Medline, EBSCOhost, and PubMed electronic databases. The eligibility criterion was research published in English between 2015 and 2023. The inclusion focused on the use and performance of telephone triage services and system-focused studies.

**Results:**

A total of 1098 papers were screened for inclusion, with 57 papers included in our review. We identified 13 performance indicators covering all Triple Aim dimensions: waiting times, access, patient satisfaction, the accuracy of triage decision, severity and urgency of the symptoms, triage response, patient compliance with the advice given, follow-up healthcare service use, and running costs of service. We didn’t find any earlier framework covering all Triple Aim dimensions properly.

**Conclusions:**

Measuring the performance of telephone triage requires an extensive and comprehensive approach. We presented performance indicators that may be included in the framework for measuring the performance of telephone triage to support overall performance measurements of telephone triage.

## 1. Introduction

Patients in emergency care are an unselected group of patients, ranging from those with acute conditions requiring acute medical care to those with non-urgent conditions who do not require immediate medical attention. Non-urgent emergency visits involve patients whose symptoms do not indicate an immediate need for treatment or treatment initiation within 24 h. These visits can be effectively managed in less acute settings during regular working hours. Many countries are working to address growing demands and control patient flows in emergency care by establishing national telephone triage numbers. Many countries are working to address growing demands and control patient flows in emergency care by establishing national telephone triage numbers. Telephone triage is a way to address growing demands and control patient flows in emergency care [1–3]. Patients must call the telephone triage services before seeking medical attention at emergency clinics. During the triage assessment process, specially trained call handlers assess the patients’ health status and urgency of the health problem and allocate the patient to the most appropriate service [2–6]. The critical task is timely patient assessment and achieving positive care outcomes.

Performance measurement in the healthcare environment is challenging but of pivotal importance in improving service quality [7,8]. Healthcare organizations are constantly looking for innovative ways to achieve a more efficient, effective, and sustainable system. A wide range of metrics and concepts have been created to evaluate the performance of healthcare systems [9–11]. One commonly used framework is the Triple Aim by the Institute for Healthcare Improvement (IHI) [12,13]. Later in 2014, Bodenheimer and Sinsky [14] suggested that the leaders and providers of healthcare should add a fourth dimension alongside the triple aim framework. The Quadruple Aim includes the goal of improving the work-life of those who deliver care because society expects more and more from healthcare professionals. In this scoping review, our primary focus was on identifying indicators suitable for external performance measurements to facilitate benchmarking using integrated and comparable data and we excluded the fourth aim as it is more important in internal development of organizations and processes.

Healthcare quality is one of the main components used for health system performance assessment [15]. Typically, healthcare quality has been measured in six dimensions – safety, effectiveness, access, patient-centredness, appropriateness, and continuity of care [12]. New methods to quantify the value of healthcare are needed to transition from volume- to value-based reimbursement [15]. However, it is not unproblematic to define what is meant by outcomes and how to measure them in the best way [16]. According to Michael Porter’s well-known equation for value assessment, value is defined as ‘the outcomes that matter to patients and the costs to achieve those outcomes’ [17].

The literature has focused on certain performance aspects of telephone triage, such as experience of service [2,18], quality of communication [2,4,19–23], safety [2,5,19,24–29] or efficiency [5,19,20]. In addition, four literature reviews have been published [18,19,24, 26]. Boggan et al. (2020) evaluated the effects of remote triage systems on healthcare utilization, case resolution, and patient safety outcomes [24]. Sexton et al. (2021) focused on evaluating telephone triage from the viewpoints of service user experience, service use, and clinical outcomes [18] describing the characteristics of patients accessing telephone triage services, the type of advice received, and the patient’s telephone triage experience. Gustafsson and Eriksson (2020) identified factors that indicate quality in telephone nursing. The study focused heavily on indicators of the quality of service. The authors found factors that indicate quality in telephone nursing, namely the availability and simplicity of the service, sustainable working conditions, call-handler education and experience, healthcare resources and organization, good communication, person-centredness, competence, correct and safe care, satisfaction, and efficiency [19]. Lastly, Lake et al. (2017) conducted an overview of telephone triage studies to determine the scope, consistency and generalizability of findings to telephone triage governance, safety, and quality. Their review shows that the available evidence does not provide definitive answers to questions about the quality of care provided, access and equity of the service, its costs, and outcomes [26]. All of the literature review articles were written from a precisely delimited and chosen perspective, and hence the previous studies still need to be evaluated more comprehensively to identify the critical indicators of the overall performance measurement. Studies or frameworks focusing on overall performance are more difficult to find.

The purpose of this article is to summarize indicators used in telephone triage services research and to make recommendations for the selection of valid indicators to measure the performance of telephone triage. We used the Triple Aim framework to identify indicators to measure the overall performance of telephone triage. The Triple Aim framework consists of improving the patient care experience, improving populations’ health, and reducing cost per capita. The objective was to find measures for each Triple Aim dimension. We describe what kind of frameworks, performance indicators, or variables have been used for evaluating telephone triage performance by systematically mapping telephone triage performance measurement. We aim to find a comprehensive framework including all performance dimensions to measure the overall performance of telephone triage. The research question we seek to address is: What kind of frameworks has been used to measure the performance of telephone triage, and what are the performance indicators? To this end, we will define the indicators required to measure the performance of telephone triage using the scoping review method.

## 2. Methods

Performance measurement refers to the measurement of the outcome, results, efficiency and effectiveness at different levels of organizations [17]. It is pivotal to identify mechanisms by which a service achieves value and to be able to link indicators at different levels [30]. A typical way of measuring overall performance is to use a performance measurement framework covering relevant dimensions. The Triple Aim [13,14], based on the idea of health system improvement by simultaneously pursuing three dimensions: the health of populations, the patient experience of care and satisfaction with service, and reducing the per capita cost of healthcare. All dimensions should be simultaneously developed and balanced to achieve a high-quality healthcare system [11, 13, 31]. We used the Triple Aim framework in our study, but as we measure a specific service instead of the healthcare system as a whole, we replaced population health with health outcomes. After grouping the indicators, we used Donabedian’s (1980) classification of strategic indicators. According to Donabedian’s conceptual model, KPIs can be classified by being related to the three components of the healthcare system: structures, processes, and outcomes [32]

### 2.1. Study design

In this scoping review, we followed the protocol in the Joanna Briggs Institute (JBI) scoping review manual. We mapped and identified which frameworks, KPIs and variables have been used for telephone triage performance measurement concepts and duly clarified which independent KPIs or frameworks have been used in earlier studies [33]. The choice of method was based on the fact that the phenomenon under study is versatile, coupled with the fact that new discoveries are constantly being made. Our goal of mapping concepts essential for further engaging information users when synthesizing information also supported the choice of the scoping review method [33].

### 2.2. Information sources, search strategy and study selection

We selected the CINAHL, Medline, EBSCOhost, and PubMed databases, and included articles published in peer-reviewed journals relevant for measuring the sustainability performance of telephone triage services. Searches were performed by one reviewer (HV) between 22 March 2021 and 13 Jun 2023. The results were discussed within the research group in each phase of the study. The inclusion criteria for the articles were the publishing year (2015–2023), and the use of English as the publication language. Our search included the following key words: (telephone AND (triage OR consult* OR out-of-hours*) ((telephone) AND (urgency)) AND (assessment), ((Telephone triage) AND (Telephone assessment), ((telephone) AND (triage AND consult)), ((Telephone) AND (pre triage)); ((Telephone AND (triage AND performance).

One researcher (HV) took the lead in data extraction, and the other researcher (PT) reviewed the search and validation process. After the initial search, the duplicate records were removed (*n* = 266). Based on the title and abstract screening, we excluded articles related only to telephone triage for a specific medical health problem or patient group and if the articles that examined a specific triage classification or its validity without a usability evaluation of telephone triage (*n* = 1898).

The inclusion criteria focused on the use and performance of telephone triage services and on system-focused studies. The search process is described below using the Prisma flowchart, and the search results are reported according to the PRISMA-ScR checklist (Supplementary Appendix A). The 308 reports were sought for retrieval. The 133 reports not retrieved based on the reading of the abstract were excluded if the study abstracts needed more critical information about the study setting or methodology. Also, if the study setting was something other than an emergency or urgent care or the addressed topic was not telephone-based triage, the study was excluded.

We reviewed and evaluated the articles that were included through three Triple Aim framework dimensions. The measurement framework was subsequently constructed, combining results from the literature review and the Triple Aim framework. The objective was to find measures for each Triple Aim dimension.

## 3. Results

We identified 13 performance indicators covering all Triple Aim dimensions: call duration, waiting times, access, patient satisfaction, patient’s experience, the accuracy of triage decision, severity and urgency of the symptoms, triage response, patient compliance with the advice given, follow-up healthcare service use, hospitalization, ED visit outcomes and running costs of service. Two dimensions were divided to subdimensions and the final framework has the dimensions of: Service accessibility, Patient opinion on the quality of service, Effectiveness and safety, Patient-relevant outcomes and Costs per case treated.

### 3.1. Article identification and selection process

**Figure 1.**
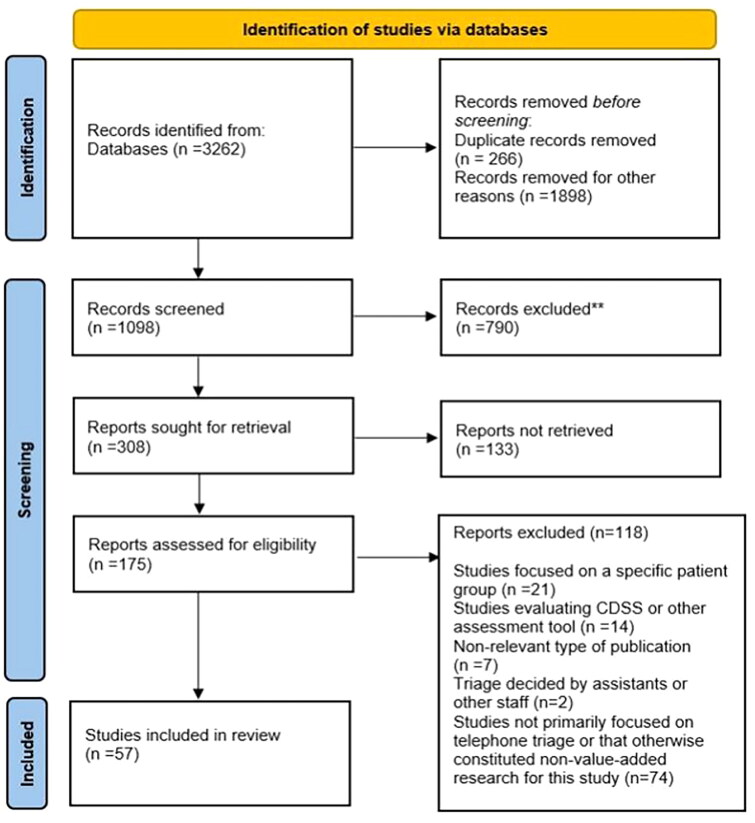
The PRISMA flowchart used in the scoping review.

The database literature search yielded 2 996 records after deduplication (Figure 1). In the end, we assessed 175 records, 57 of which were included in the scoping review. A summary of the included articles and their study designs (*n* = 57), aims, indicators and key findings from the perspective of the Triple Aim framework can be found in SupplementaryAppendix B. We excluded studies focused on a specific patient group, studies that evaluated CDSS or other assessment tools, studies that were non-relevant types of publication, studies not previously on telephone triage or that otherwise constituted non-value-added research for this study, such if the triage not in the prehospital settings. The included articles were methodologically diverse, representing observational studies (*n* = 15), cohort studies (*n* = 8), mixed methods studies (*n* = 6), cross sectional studies (*n* = 6), randomized trials (*n* = 5), descriptive studies (*n* = 5), reviews (*n* = 4), quasi-Experimental studies (*n* = 3), comparative studies (*n* = 2), economic evaluation (*n* = 2) and validation study (*n* = 1). Most articles originated from Denmark (*n* = 19). Ten studies originated from the United Kingdom, seven from Australia, five from the US, three from Belgium and Sweden, two from the Netherlands and Ireland, and one study each from New Zealand, Austria, Brazil Finland, Japan, and Norway.

**Figure 2.**
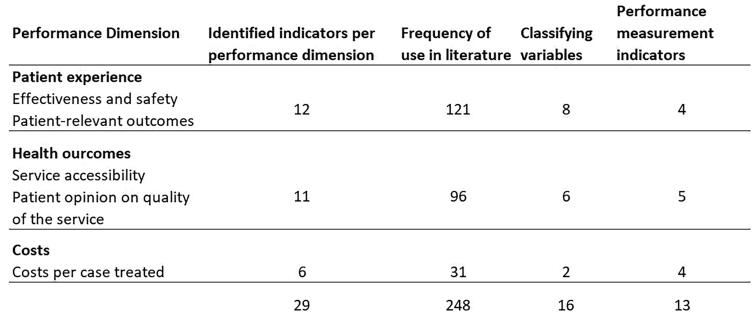
Indicator groups based on the IHI triple aim dimensions.

After reviewing the studies, they were categorized based on their objectives and purpose, after which we proceeded to review and classify the individual indicators. The indicators were identified based on the scoping literature review and categorized into three Triple Aim dimensions (Figure 2).

### 3.2. First dimension: experience of care

Table 1.Indicators of the Triple Aim dimension: Patient experience of care

#### 3.2.1. Service accessibility

Telephone triage services offer quick and easy access to advice and help when required [26, 36] and the primary motivators for calling are concerns about one’s own or a family member’s health [1, 25]. The contact volumes change over time. Siddiqui et al. (2020) recognized that KPI analysis related to contact times should be incorporated into future research with a view to understanding, predicting and modifying consumer behaviour [67].

In addition, the characteristics of users vary according to when they are contacted, the type of contact, and the frequency of contact [3, 37]. Elliot et al. (2015) found that there were significant differences in the types of problems presented during normal hours and out of hours. They also reported differences in the duration of symptoms between contacts at different times. Most problems (62.9%) had lasted <24 h before the call, but out-of-hours calls tended to be for problems of a shorter duration [47] Furthermore, service availability and waiting times are related to patient experience and service satisfaction [55].

#### 3.2.2. Patient opinion on quality of service

A patient’s opinions on the quality of service are crucial to the acceptance and utilization of the service, and also influence adherence to the advice given [27]. Moreover, to strengthen the overall efficiency, information on the system and processes is needed, with one aspect being patient satisfaction with the service [56]. In Zinger et al.’s (2019) study on caller satisfaction, patient satisfaction was associated with waiting time, the reason for the encounter, and triage response. Poor experiences of system functioning are reflected in outcome indicators, including utilization of service and patient’s health outcomes. Telephone triage’s influence on patients’ care-seeking behaviour positively affects the health system when it recommends appropriate care. Patients’ compliance with advice was associated with patient satisfaction with the service, the type of helpline advice received, and the estimated severity level of the conditions [56] Improving patient satisfaction with the service and understanding of the advice can increase compliance [27]. An analysis of patient satisfaction provides information on whether interventions lead to better outcomes and whether these improve the quality of services in healthcare systems [56]. Wallace et al. (2018) aimed to characterize patient complaints in an out-of-hours general practice setting. They looked at complaints about the following variables: the education of professionals, the rate of patient complaints, and the type of complaints. Most of the complaints were related to clinical care problems such as diagnosis and prescribing. Common themes were also unmet management expectations and clinical examination dissatisfaction [41]. The quality of communication has also been associated with the appropriateness of the assessment of urgency and required care, and thus with the safety of triage as well [2,4,20].

### 3.3. Second dimension: health outcomes

Table 2 Indicators of the Triple Aim dimension: Health outcomes.

#### 3.3.1. Effectiveness and safety

Patients have broadly varied symptoms, conditions and issues when they contact telephone triage services [42,53]. Many of the previous studies used reason-for-encounter indicators from different perspectives. Moth et al. (2016) described the reasons for encounters, the applied diagnoses, and the severity of health problems presented in calls, and used the respective indicators. They stated 392 different reasons for encounter out of 7,810 telephone contacts, of which the potentially severe category made up the core (52.3%) [42].

Efficient and safe telephone triage requires a balance between having a minimum of under-triage and keeping over-triage at an acceptably low level. Graversen et al. (2019) developed the 24-item assessment tool, which focused on measuring patients’ experiences, particularly from a communication perspective, and assessing overall safety in telephone triage. Poor experiences of system functioning are reflected in outcome indicators such as utilization of service and patient’s health outcomes [2].

Nørøxe et al. (2017) described the frequency and characteristics of medically inappropriate calls and examined the severity of the patient-assessed problems compared to the medical inappropriateness of the contact. They found that one in four calls was medically inappropriate. Those contacts were associated with younger age, longer symptom duration, exacerbation of chronic condition, and contact only a few hours outside the patient’s own GP’s office hours. Medically inappropriate contacts related to symptoms lasting over 24 h concerned exacerbation of a chronic condition. For over half of the inappropriate contacts, the health problem was considered severe by patients still associated with unfulfilled patient expectations [46].

An accurate assessment is also a challenge due to limited knowledge of the patient, a higher likelihood of severe illness, and time pressure [5]. On average, 10% of all contacts are potentially unsafe because of the level of urgency or type of care required being underestimated [5, 28, 71]. In a study by Smits et al. (2016), critical care was assessed adequately in 63.6% of cases, over-estimated in 19.3%, and under-estimated in 17.1% [6]. Lake et al. (2017) found that approximately 50% of calls could be handled with telephone advice alone, meaning that the patient is not referred further to a healthcare unit [25]. In Huibers et al.’s (2016) study, A total of 59% of all calls ended with a telephone consultation [3]. Inadequate communication and a non-normative symptom description contributed to under-triage [71]. Triage of patients to an adequate care level is successful if protocols, flow charts, and care levels are well defined and the call handler is well trained [29].

#### 3.3.2. Patient-relevant outcomes

The critical outcome indicator is whether the patient received the help they needed. The main dimensions of efficiency, such as accessibility of service and use of services, are indicators that could potentially improve service quality and effectiveness. Triage tools are criticized for failing to incorporate the patient perspective and context [34]; the same is true of viewpoints on the assessment of patient-relevant outcomes, which have rarely been singled out as an independent whole in previous studies.

The value of the service is generated when care is resource-efficient and suitably urgent, and callers follow the advice given. Patients seeking services that are inappropriate for the situation degrade healthcare outcomes due to inefficiency, congestion, and increasing dissatisfaction among both staff and patients. Patient compliance is an outcome of telephone triage and demonstrates the effectiveness of the service [67]. Non-compliance with advice when seeking appropriate care leads to poorer health outcomes [66]. Gibson et al. (2018) provided evidence whereby 66.5% of patients were compliant with a disposition to attend an ED after telephone triage [43]. Li et al. (2016) added that patients who were satisfied with the service were more likely to follow the advice given [27].

### 3.4. Third dimension: reducing the per capita cost

Table 3.Indicators of the Triple Aim dimension: Reducing the per capita cost of healthcare.s

Siddiqui et al. (2020) studied how telephone triage services influence the wider health system. They considered that the service is related to the significant dynamics of indicators: access to care, compliance, response to instructions, patient satisfaction, cost, safety, physician workload, and clinical outcome. Extensive inappropriate service use can lead to poorer health outcomes, inefficiencies, overcrowding, frustrations, and complaints. A telephone triage to improve patient care and health system functioning will require a greater understanding of the patient cohort and the factors that shape non-compliance and compliance with triage recommendations [67].

From a service system perspective, telephone triage promotes both clinical and functional integration, helps manage patient flows, and reduces costs, with patients being directed promptly to the appropriate intensity of care [48, 52, 67]. There is no consensus on how telephone triage services broadly affect the demand for healthcare services or whether they reduce the workload in emergency units, and the state of knowledge about the effects of telephone triage on ED use is conflicting. Howell et al. (2016), Knowles et al. (2016), McKenzie et al. (2016), and Gibson et al. (2018) found that telephone triage may reduce ED attendance [39, 43, 57, 64]. Howell et al. (2016) investigated the relationship between telephone triage and ED visits. They found that telephone triage decreased visit volume in the ED in a clinically and statistically significant way [64]. The authors concluded that a telephone triage service could help decrease ED crowding, especially by communicating other care options to patients with low acuity health problems. These findings indicate the clinical significance of a telephone service in reducing ED use.

Knowledge of telephone triage effects on other service use is conflicting. Roivainen et al. (2020) found that telephone triage reduced non-urgent EMS admissions by one-third [44]. Holt et al. (2016), in contrast, examined whether telephone triage reduces clinician-patient contact time on the day of the request, compared with usual care. They found that telephone triage is not associated with a reduction in overall clinician contact time during the index day [53]. Ostermann et al. (2019) assessed the impact on health service demand after patients had consulted the telephone-based system. They used the conceptual model of ‘‘shift cases’’ from one particular service setting, such as an outpatient clinic, to another to calculate savings realized through patient shifts. The authors identified threshold values for cost-effective operation based on potential savings in private and public costs and running costs. The calculated costs varied depending on the capacity utilization rate. A financial advantage will be achieved if service users can be directed to sufficiently stable resources in a correspondingly lower-cost care environment. The final economic benefits are dependent on how patients observed the instructions provided and the cost of organizing services. The authors concluded that telephone-based triage systems are a potentially cost-effective strategy to reduce avoidable encounters. The public savings of a shift in the provider setting vary depending on the level of service delivery, with diminishing savings for shifts at lower levels [58].

### 3.5. Performance framework for telephone triage services

**Figure 3.**
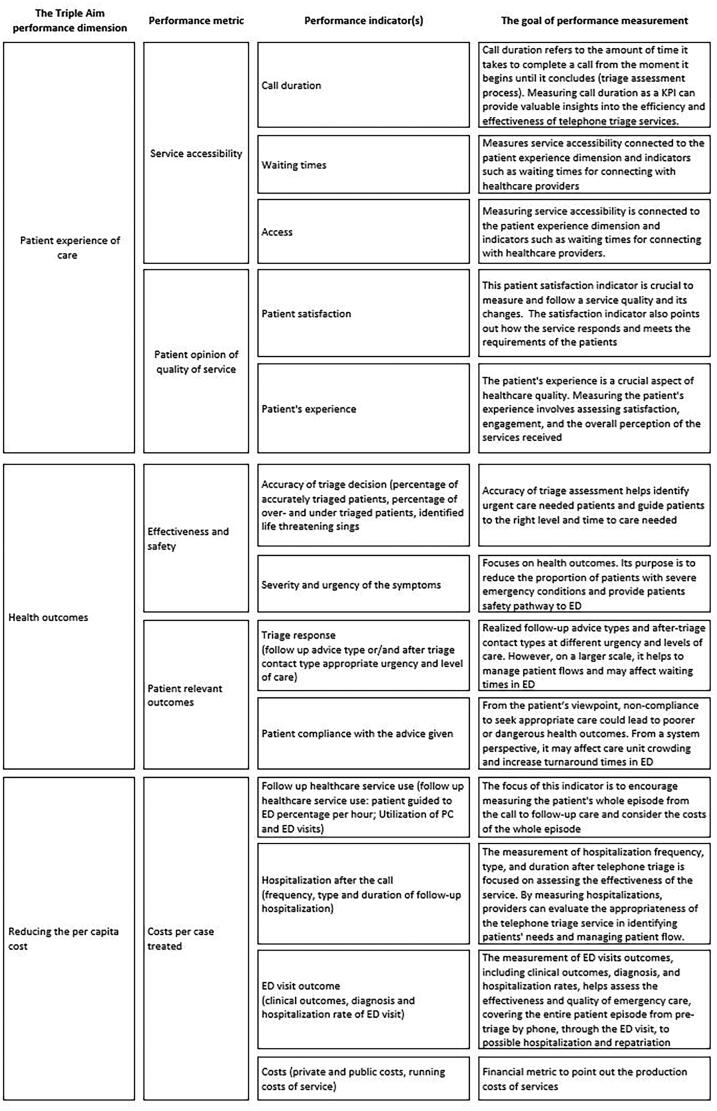
Performance framework for telephone triage services, based on the scoping review.

From all identified indicators, we found 13 indicators to measure the performance of telephone triage services. Based on this research, the following (Figure 3) five performance aspects and 13 indicators can be considered compatible with the Triple Aim framework to measure the performance of telephone triage services. We also illustrate the significance of the use of the individual indicators from the viewpoint of performance measurement as a whole.

## 4. Discussion

In this study we comprehensively identified and summarized the performance measurement indicators from the Triple Aim perspective using the scoping review method. A key finding was that there was no general overall performance measurement framework to measure the performance of telephone triage. Still, the need for comprehensive, acceptable and viable performance indicators set to underpin a measurement framework is emphasized in the literature [12,13, 17].

The findings from the 57 retrieved articles showed that no previous measurement framework included all dimensions of the Triple Aim framework. In addition, process and structural metrics were emphasized in the classification in previous studies instead of outcome metrics, excluding the dimension of reducing the per capita cost (Table 1). However, the cost dimension differs from the other dimensions significantly in that the costs were included in the frameworks significantly less often than the other performance indicators. There were far fewer studies measuring per capita costs; only Ostermann et al. (2019), Siddiqui et al. (2020) and Rebolho & Raupp (2023) paid attention to the cost effects of telephone triage services [52, 58, 67]. If we look at the study results from the point of view of individual indicators, typically the indicators used measured the health outcomes, mainly from effectiveness and safety perspectives (Table 2). In contrast, overall, the indicators to measure cost were not conceptually as straightforward as the indicators to measure patient experience or health outcomes (Table 3). A measurement framework encompassing all performance dimensions with balanced representation across process, structural and outcome indicators is considered to be a key element of health care performance measurements and improvement of services performance [12]. Measuring per capita costs is a challenge, but it requires that we work to capture all relevant expenditures to measure costs in a care system and achieve high value for patients. As Porter said, the goal of healthcare delivery needed to be defined with value as the health outcomes achieved per dollar spent [12,17].

**Table 1.** Indicators of the triple aim dimension: Patient experience of care.

Performance dimension: Patient experience of care	Frequency	Indicator category	Classifying variable	Performance measurement indicator	References
Service accessibility					
Call arrival time *(start and end time of call; time in relation to own health centre)*	22	Process	x		[20,21,25,29,34–51]
Total volume of calls	23	Process	x		[1,20–22,29,36,37,39,41,43,45,46,52,54–61]
Call duration	9	Process		x	[5,20–22,40,41,45,59,62]
Type of call *(callbacks, direct calls, administrative contacts, medication-related calls)*	4	Structural	x		[4,45,61,62]
Waiting times	4	Process		x	[25,35,51,63]
Access	1	Process		x	[64]
Patient opinion on the quality of service					
Patient satisfaction	11	Outcome		x	[2,25,38,41,45,50,51,63–66]
Communication *(professional communication skills and quality of communication)*	8	Structural	x		[1,2,4,5,21,22,38,52]
Complaints *(rate and type)*	1	Process	x		[67]
Patient’s experience	3	Outcome		x	[2,18,38]
Professional competence	10	Structural	x		[4–6,22,29,46,58,60–62]

**Table 2.** Indicators of the triple aim dimension: Health outcomes.

Performance dimension: Health outcomes	Frequency	Indicator category	Classifying variable	Performance measurement indicator	References
Effectiveness and safety					
Reason for encounter (as in ICPC-2)	27	Process	x		[1,3,5,21,22,25,28,29,34,38,42–47,49,53,59–61,65,68,69,71]
Accuracy of triage decision *(Percentage of accurately triaged patients, percentage of over- and under-triaged patients, identified life-threatening signs)*	11	Outcome		x	[3,5,20,28,29,35–37,42,45,46,50,65,66,69,71]
Severity and urgency of the symptoms	13	Outcome		x	[3,25,27,28,42,46,55,60,61,66,69,72]
Duration and development of patient symptoms	10	Process	x		[3,20,35,40,42,46,47,65,66,69]
Co-morbidity	6	Structural	x		[1,34,42,46,55,66]
Type of current medical problem	3	Structural	x		[41,45,69]
Cause (possible) and consequence of symptoms	1	Structural	x		[35]
Patient-relevant outcomes					
Triage response *(follow-up advice type and/or after-triage contact type, appropriate urgency and level of care)*	36	Outcome		x	[1,3,5,20,21,25,27–29,34,36,37,41,42,44,46,47,49,50,53,55,56,58–70,72]
Patient compliance with advice given	8	Outcome		x	[21,27,49,52,59,62,67,68]
ICD-10 diagnoses	3	Structural	x		[34,60,71]
Mortality	1	Outcome	x		[70]
Patient medical history and medications	2	Structural	x		[5,59]

**Table 3.** Indicators of the triple aim dimension: Reducing the per capita cost of healthcare.

Performance dimension: Reducing the per capita cost	Frequency	Indicator category	Classifying variable	Performance measurement indicator	References
Costs per case treated					
Follow-up healthcare service use *(patient guided to ED percentage per hour; utilization of PC and ED visits)*	10	Outcome		x	[34,39,41,43,50,57,59,61,70,72]
Number of calls to telephone triage per patient by specific time	5	Process	x		[26,36,37,50,52]
Hospitalization after the call *(frequency, type and duration of follow-up hospitalization)*	5	Process		x	[1,47,57,70,73]
Service effects on healthcare utilization *(number of office visits to primary care clinics/ED)*	4	Structural	x		[39,43,47,70]
ED visit outcome *(clinical outcomes, diagnosis and hospitalization rate of ED visit)*	4	Outcome		x	[42,52,55,64]
Costs *(private and public costs, running costs of service)*	3	Outcome		x	[48, 64,71,72]

Performance measurements, including process and outcome measures, play a pivotal role in the current healthcare environment. Information about health service performance is needed to support decision-making, and to measure and improve the quality of services. It is essential to empower telephone triage service providers to identify the crucial performance indicators to follow, report and benchmark with other providers’ performance metrics. In addition, telephone triage personnel also need to understand what is expected and what kind of entities are being measured in respect of their work or its outcomes in order for them to receive support in committing to measurable goals.

The current frameworks have measured the performance of telephone triage from some specifically limited viewpoints, and the previous literature did not present a framework for measuring overall performance. Therefore, we suggest that performance measurement should be pursued at different levels of the system by measuring the service system’s capacity with reliable methods and suitable performance indicators. Our framework encapsulates used concepts and indicators seen in previously published studies. The framework provides a means to resolve typical challenges related to the choice of indicators by identifying 13 clearly defined indicators that comprehensively synthesize 57 studies of telephone triage performance.

### 4.1. Strengths and limitations of the review

One of the strengths of this study lies in the comprehensive literature search and the use of multiple well-known databases. On the other hand, the methodological weakness of the study is related to the nature of the scoping review. Such reviews do not rate the standard of the evidence, which can lead to including studies of questionable quality in the research. Moreover, the methodological choices may give rise to limitations. For example, restricting the search to studies published between 2015 and 2023 may have resulted in some earlier articles being overlooked. However, the inclusion of earlier frameworks [2, 5] ensures that previous literature has also been taken into account. As telephone triage services have expanded and become a recognized part of the acute care services in recent years, this inclusion criterion was deemed appropriate. The second methodological limitation is that one reviewer abstracted the data, which were then verified by other reviewers.

Furthermore, it is important to acknowledge the limitations of our findings. Firstly, our study is based solely on scoping reviews of existing literature. Secondly, the study results rely on indicators used to measure performance in telephone triage in the literature, and these have not been comprehensively tested in practical applications. Thirdly, we did not engage healthcare industry experts in the process of selecting indicators and evaluating how these selected indicators could be practically applied to measure performance in telephone triage. Therefore, from a practical perspective, it will be necessary in the future to involve professionals in the final indicator selection process. To this end, we recommend further research in which the measurement framework is tested in real healthcare environments.

One key limitation of our results that needs to be taken into account when applying the results in different settings, such as between telephone triage in primary care and emergency care, is that the criteria for a successful telephone triage may vary depending on the context in which the triage is performed.

## 5. Conclusion

Measuring the performance of telephone triage requires an extensive and comprehensive approach. In this scoping review, we identified and presented performance indicators that may be included in the framework for measuring the performance of telephone triage to support overall performance measurements of telephone triage. Our study has both academic and practical implications. From an academic viewpoint, the study forms the basis for future research on measuring the performance of telephone triage in a healthcare organization. From a practical viewpoint, we have uncovered valid indicators for the future development of reports that support performance measurement and opportunities for benchmarking based on integrated and comparable data.

## Supplementary Material

Supplemental Material

Supplemental Material

## Data Availability

No unpublished data are available following this study.

## Patient involvement

Patients were not involved in the design.
